# Twisted Bands with Degenerate Points of Photonic Hypercrystals in Infrared Region

**DOI:** 10.3390/nano12121985

**Published:** 2022-06-09

**Authors:** Yaoxian Zheng, Qiong Wang, Mi Lin, Luigi Bibbò, Zhengbiao Ouyang

**Affiliations:** 1 THz Technical Research Center of Shenzhen University, Shenzhen Key Laboratory of Micro-Nano Photonic Information Technology, Key Laboratory of Optoelectronic Devices and Systems of Ministry of Education and Guangdong Province, College of Physics and Optoelectronic Engineering, Shenzhen University, Shenzhen 518060, China; jewel5282@163.com (Y.Z.); qwang@szu.edu.cn (Q.W.); linfengas111@szu.edu.cn (M.L.); 2 Department of Information Engineering, Infrastructure, and Sustainable Energy (DIIES), Mediterranea University of Reggio Calabria, 89124 Reggio Calabria, Italy; luigi.bibbo@unirc.it

**Keywords:** photonic crystals, hyperbolic metamaterials, photonic hypercrystals, self-collimation, switching

## Abstract

Photonic hypercrystals (PHCs) are materials combining hyperbolic metamaterials (HMMs) with widely used photonic crystals. We found that finite-sized Type-I HMMs can support unique electromagnetic modes, which could be utilized in two-dimensional photonic crystals to achieve PHCs with twisted bands in the infrared region. Numerical investigation of the PHCs showed that the twisted bands have degenerate points that can support all-angle self-collimation effects. The behaviors of light beams change dramatically in such bands, which provides an effective method in controlling light propagation and can be applied as switching. The effect of the filling factor and the permittivity of the dielectric medium of the HMM on the twisted bands were studied. Furthermore, by considering the nonlinear effect of the dielectric layers, an all-optical switch working on the PHC twisted bands is proposed, which has low switching power and high extinction ratio (19.75 dB), superior to conventional HMM switches that require type transformation of metamaterial.

## 1. Introduction

Hyperbolic metamaterials (HMMs) are a particular kind of materials with anisotropic dielectric tensors [1,2,3], which show unprecedented ability in controlling the propagation of electromagnetic waves. With hyperbolic curves in the wave vector space, HMMs can support unique electromagnetic modes with excellent performance, and, thus, have become advanced building blocks for photonics [4,5,6,7]. The study of HMMs has made great advances, and many exciting phenomena of this unusual type of material have been discovered [8,9]. Based on the signs of the transverse and longitudinal components of the effective permittivity (denoted as $\varepsilon_{⊥}$ and $\varepsilon_{∥}$, respectively), HMMs can be sorted, according to their performances, as: (i) Effective dielectric ($\varepsilon_{⊥}>0,\;\varepsilon_{∥}>0$), (ii) Effective metal ($\varepsilon_{⊥}<0,\;\varepsilon_{∥}<0$), (iii) Type-I HMM ($\varepsilon_{⊥}>0,\;\varepsilon_{∥}<0$), and (iv) Type-II HMM ($\varepsilon_{⊥}<0,\;\varepsilon_{∥}>0$). The transformation between two different types of HMMs can be utilized as optical switching applied in wave-propagation control systems [10,11]. For instance, the effective refractive index of a Type-I HMM can be negative, and such an HMM only supports wave vectors opposite to the direction of energy flow. However, in an HMM that performs as Effective metal, no transmitted modes can be supported, resulting in total reflection of incident light. On the other hand, photonic crystals (PCs) are a type of artificial materials with periodic distribution of permittivity [12,13]. By utilizing the gaps of band structures, it is possible to obtain optical cavities, waveguides and other components. Moreover, PCs have abundant dispersion properties. Many interesting phenomena, such as negative refraction, super-prism effect and slow light effect, have been explored in research work [14,15]. Metamaterials and PCs have structures with similar concepts. Researchers have made great efforts to bring them together. One example is a kind of novel optical material named photonic hypercrystals (PHCs) [16,17,18]. PHCs can have new properties, such as omnidirectional band gap [19] and Goos-Hanchen shift [20], which show increasing potential in achieving devices with good performance. On the other hand, in the PHC system, the properties of HMMs can also be modified by PCs [21], which could bring new phenomena.

Extant studies mostly focused on the type of transformation of HMMs [9,10], as well as continual discovery of novel properties of HMMs [16,17,18,19,20]. The property of two-dimensional (2D) PHCs has not yet been studied adequately. However, 2D structures are clearly more essential for integrated optics, especially when considering the fabricability for various applications. The self-collimation (SC) effect is one of the dispersion properties of 2D PCs, where wave propagation remains in one direction. The all- angle SC effect is one of the SC effects that can support incident lights from every direction. In previous work, we have applied HMMs in PCs to enhance the SC effect [21]. Here, we take a step further, to explore the property of PHCs in the infrared region, where the HMMs are Type-I HMMs with opposite signs of permittivity in different directions. We found that PHCs in the infrared region exhibited unique properties and form twisted bands with degenerate points.

The twisted bands of PHCs originate from the interaction of the modes of the finite-size Type-I HMM rods and the lattice of the PC. In the infrared region, PHCs with Type-I HMM rods have many flat bands [22], which are due to the high absolute value of the negative transverse components of the effective permittivity of the HMM. The modes in flat bands indicate strong localization, but huge material absorption may occur. Modes of another category form the twisted bands. They are unique modes generated by the periodic distribution of HMM rods. The twisted bands have some particular points that support the all-angle SC effect, indicating large potential in the modification of beam behavior in the PHC. In fact, simple HMMs based on the dielectric/metal multilayer structure, or metal wire array structure, can also achieve SC [23], but type transformation is required and the efficiency is low. The formation of twisted bands expands our understanding of the modes of finite-size Type-I HMM, and the PHC in the infrared region also has advantages by comparison with previous ones [21]: (i) the twisted bands provide abundant modes for changing beam behavior. For instance, when increasing the operating frequency, the beam behavior changes from divergence to collimation, then to convergence and to collimation again, and finally to divergence. The PHC with such a twisted band could be utilized as versatile tunable lens; (ii) PHCs with Type-I HMM rods have flat bands, which results in the total reflection of incident light on the boundary (due to the mismatch of wave vectors). On the other hand, SC modes of PHCs have high transmissions. Thus, by changing the working states of PHCs, from modes in flat bands to SC modes, optical switches with high efficiency can be obtained. The extinction ratios of the switches could reach very high, being much more effective than the previous ones.

In this paper, the unique twisted bands of PHCs in the infrared region have been investigated, and, based on the results, optical switches are proposed. The PHC systems have combined the concepts of HMM and PC. Dispersion properties of the PCs have been modified by the HMM rods, with the enhancement of field localization, which is essential in increasing tunability. Compared to the conventional method of utilizing the transformation of HMM types, PHC switches can provide more efficiency in integrated photonics.

## 2. Materials and Methods

**Figure 1:**
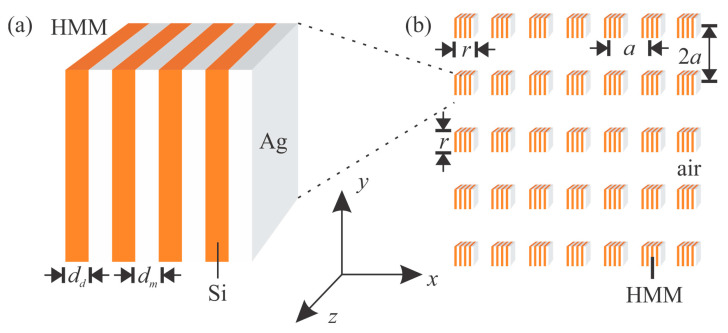
Sketch of (**a**) an HMM consisting of Si and Ag layers, and (**b**) a 2D PHC containing HMM rods.

The proposed 2D PHC consists of HMM square rods in the background medium of air (n_0_ = 1), and the structure parameters and operating frequencies are different from that reported in Ref. [21]. As shown in Figure 1, the lattice constants of the PHC in x and y directions were a = 1 μm and b = 2a = 2 μm, respectively, and the side length of the HMM rods was r = 0.6a. The HMM rods consisted of metal (Ag) and dielectric (Si) layers. The thicknesses of the Ag and Si layers were d_m_ = 80 nm and d_d_ = 120 nm, respectively. Thus, an HMM rod contained 6 layers, and its filling factor (of metal) was 0.4. The 2D PHC was investigated by using the plane-wave expansion method and finite-element method, and the commercial software COMSOL Multiphysics (COMSOL Inc., Burlington, MA, USA) was used. Only TM waves (H_z_ polarization) were considered, and the light waves were propagated in the x-y plane ($\varepsilon_{⊥}=\varepsilon_{xx}$, $\varepsilon_{∥}=\varepsilon_{yy}=\varepsilon_{zz}$). Note that another experimental scheme, other than that in Figure 1, might be more practical [24], in which case, the polarization and wave vector should be changed accordingly.

Experimental data of the permittivity of Si was used in the simulation [25,26]. The refractive index and extinction coefficient of Si were n_Si_ = 3.4870 and k_Si_ = 0.0000 at the frequency of 200 THz, respectively. In order to be more universal, the permittivity of Ag was described using the Drude model [27], as:

(1) $$\varepsilon_{\mathrm{Ag}}=\varepsilon_{\infty }-\frac{\omega_{p}^{2}}{\omega (\omega +i\gamma )},$$

where $\varepsilon_{\infty }$, $\omega_{p}$, γ, and ω are the high-frequency permittivity, the plasma angular frequency, the damping term, and the operating frequency, respectively. From the experimental data of the permittivity of Ag [28,29], terms in Equation (1) can be obtained as $\varepsilon_{\infty }$ = 1.4447, $\omega_{p}$ = 1.328 × 10^16^ rad/s and γ = 9.1269 × 10^13^ rad/s. We also compared the differences between the experimental data and the Drude model data of permittivity, and found that the Drude model can describe the permittivity of Ag very well, as the data from the Drude model was very close to experimental data. Note that the chosen material can be flexible, because various materials can be applied in the hyperbolic metamaterial, and twisted bands with degenerate points can still be obtained. This is because the twisted bands of the PHC are formed by interaction between the unique permittivity tensors of hyperbolic metamaterials and the Bloch modes of photonic crystals. The reason we adopted the Drude model was that we could obtain more general results, so that the proposed model could be realized with various materials.

From Equation (1), the imaginary part of Ag is the main part of loss for wave propagation. In the infrared region, the absolute values of both the real and imaginary parts of the permittivity of Ag are very large, thus, waves cannot propagate inside bulk Ag because of its high loss and the boundary condition, which is very difficult to fulfil. As a result, the incident light will be reflected at the boundary of free space and bulk Ag. Thus, for a 2D PC system, it was important to use HMM rods rather than bulk metal rods. HMM rods can maintain the field-enhancement property of metal and provide unique modes for versatile applications.

**Figure 2:**
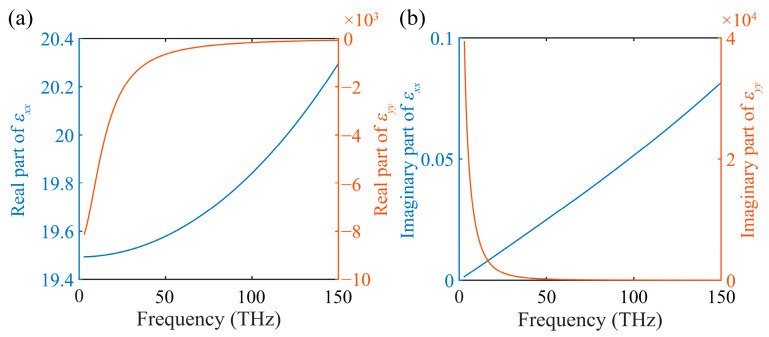
The longitudinal and transverse components of the effective permittivity ($\varepsilon_{xx}$ and $\varepsilon_{yy}$) of the HMMs. (**a**) and (**b**) show the real and imaginary parts, respectively.

Since the thickness of a metal layer or a dielectric layer is small enough for electromagnetic waves that propagate in the system in the infrared region, the effective medium method was valid (see Supplementary Materials and Figure S1) and could, thus, be applied to investigate the properties of the HMM rods. Based on the effective medium method, the HMM consisting of Si and Ag layers indicated an isotropic permittivity tensor (which could also be confirmed by numerical calculations). The permittivity components in the perpendicular and parallel directions to the incident wave vector, corresponded to the longitudinal and transverse components ($\varepsilon_{⊥}$ and $\varepsilon_{∥}$), respectively, and can be written as below [30]:

(2) $$\varepsilon_{⊥}=\varepsilon_{xx}=\frac{\varepsilon_{\mathrm{Ag}}\varepsilon_{\mathrm{Si}}}{f_{\mathrm{Si}}\varepsilon_{\mathrm{Ag}}+f_{\mathrm{Ag}}\varepsilon_{\mathrm{Si}}},$$

(3) $$\varepsilon_{∥}=\varepsilon_{yy}=\varepsilon_{zz}=f_{\mathrm{Ag}}\varepsilon_{\mathrm{Ag}}+f_{\mathrm{Si}}\varepsilon_{\mathrm{Si}},$$

where $\varepsilon_{\mathrm{Ag}}$ and $\varepsilon_{\mathrm{Si}}$ are the Ag and Si permittivity, respectively. The permittivity of Ag is obtained by the Drude model as Equation (1). Effective permittivity of the HMM was calculated, as shown in Figure 2. At an operating frequency in the mid-infrared region, the HMM performed as a Type-I HMM. Such a bulk HMM could support wave propagation with negative refractive index. However, due to the mismatch of wave vector on the boundary, the waves could not propagate inside the Type-I HMM when the wave incidence was from free space. However, for a finite-sized Type-I HMM rod, unique modes could exist with acceptable loss. Moreover, by arranging rectangle-lattice HMM rods to form a 2D PHC, unique Bloch modes could be generated. Thus, in order to overcome the limitations of modulating wave propagation in both bulk metals and bulk dielectrics, a PHC containing Type-I HMM is proposed to solve the problem by providing its own dispersion properties.

## 3. Results and Discussion

### 3.1. Unique Modes and Twisted Bands of PHCs

**Figure 3:**
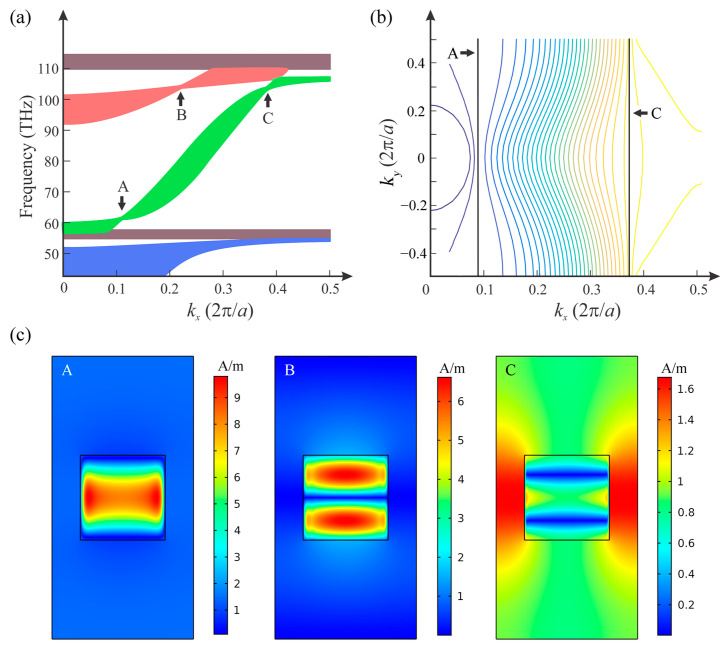
(**a**) Band structure of the 2D PHC in infrared region. Points A (59.52 THz), B (102.90 THz) and C (102.60 THz) are where dispersion curves collected, located in the third (green) and fourth (red) bands. (**b**) Equifrequency contours of the third band. (**c**) Magnetic field (|H|) Profiles of points A, B and C in a unit cell of the PC.

Figure 3a shows the band structure of the 2D PHC in the infrared region. The band structure of the PHC was calculated by using software COMSOL Multiphysics (COMSOL Inc., Burlington, MA, USA), which is based on the finite-element method. Since PHC has a repeated pattern, only one cell was needed for the calculation. Eigenvalue solver of COMSOL was used with Floquet periodicity boundary conditions to calculate eigenfrequencies. By using the parametric solver in the software to sweep the wave vector, we could calculate all the electromagnetic modes supported by the system, which allowed us to obtain the whole band structure of the PHC. The obtained bands were curved surfaces, and the three axes of the coordinate system were *k*_x_, *k_y_* and frequency. Obviously, frequency contours, as shown in Figure 3b, could, thus, be calculated based on the results. The eigenmode of a specific eigenfrequency was correspondingly obtained by the eigenvalue solver of the software, which also provided the field profile. Several flat bands and two twisted bands were found. Flat bands of the PHC contained localized modes [22]. These localized modes had large imaginary parts. Another fascinating aspect of the band structure of PHCs were the twisted bands. The twisted bands had some degenerate points, which corresponded to all-angle SC effects along the *x*-axis. For fixed *k*_x_ but different *k*_y_ corresponding to the same frequency, the dispersion curves converged at specific points.

The equifrequency contours of the third band, which is a twisted band with two SC points, are shown in Figure 3b. We can see two flat equifrequency contours of 59.52 THz and 102.6 THz, corresponding to the two all-angle SC points of the twisted band. Flat equifrequency contours indicated that Bloch wave vectors were in all directions, explaining how the all-angle SC effect occurred. On the other hand, sparse equifrequency contours meant low group velocity, and the shapes of contours changed dramatically around the SC frequency. Such a type of SC, with high frequency-sensitivity, can provide large tunability, perfectly matching the property of HMM. The beam behavior of light, which corresponded to the shape of the contour, changed dramatically in the twisted bands. For the twisted band with two SC points, when increasing the operating frequency, the beam behavior changed from convergence to collimation according to the shapes of equifrequency contours. By increasing the frequency further, beam behavior correspondingly changed from collimation to divergence until reaching the second SC point.

Nonlocality is essential for the formation of the unique band structure of the PHC, since the twisted bands with degenerate points arise as results of the spatial dispersion of HMM rods. Here, we examined the unique modes supported by the HMM rods. Figure 3c shows the magnetic field distributions of the three points in the band structure of Figure 3a. We can see that the magnetic field profiles in PCs with HMM rods are very unique by comparing them to those in PCs with conventional dielectric rods [19]. On one hand, Type-I HMMs have different longitudinal and transverse components of the effective permittivity; on the other hand, the absolute value of the longitudinal components of the effective permittivity of HMM was very large in the infrared region. Thus, in the infrared region, although incident light would be reflected by an infinite-size bulk Type-I HMM, a PHC is able to be applied in the control of wave propagation with its unique Bloch modes. Conversely, HMM rods also greatly affect the properties of 2D PCs and, thus, improve the tunability.

Besides the two SC points (points A and C) in the third band, a third SC point (point B) arose in the fourth band of the PHC, as shown in Figure 3a, which had a frequency very close to the frequency of one of the SC points in the second band. This might just have been a coincidence, as these two modes were totally different, as indicated by the mode profiles that are shown in Figure 3c. According to the profiles of A, B and C, mode A was a fundamental mode, while modes B and C were high-order modes. We could expect more high-order SC modes in the PHCs, but in higher frequencies only dense flat bands exist, and so the high-order SC modes were covered and they did not unveil themselves.

**Figure 4:**
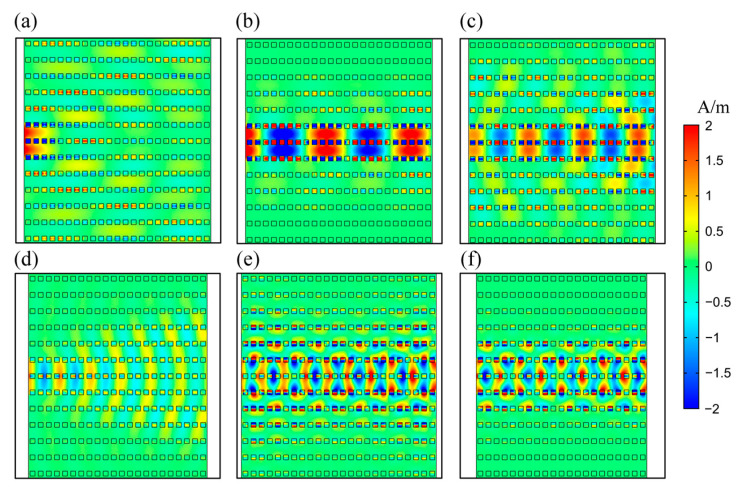
Magnetic field distributions of wave propagating in PHCs at various frequencies, with Gaussian-shape incident light located on the left. The operating frequencies of incident light are (**a**) 58.00 THz, (**b**) 59.52 THz, (**c**) 62.00 THz, (**d**) 100.00 THz, (**e**) 102.60 THz and (**f**) 102.90 THz.

Twisted bands show great potential in controlling beam behavior for the PHC system. In Figure 4, we show light propagations in the 2D PHC with different frequencies. The field distributions of wave propagation were obtained by the source driven simulation of COMSOL, which was to compute the response of the model subjected to harmonic excitations. We used different excitation frequencies, and calculated the corresponding wave propagations. The results were obtained by using frequency domain study of the software, which was also based on the finite-element method. As indicated by the band structure and equifrequency contours, beam behaviors were different at different frequencies.

Figure 4 shows the various beam behaviors of divergence, collimation and convergence. Here, the incident light was introduced to the left side of the PHC with a Gaussian shape, and the width of the beam was 2a. The field distributions clearly indicated the ability of the PHC to support various beam behaviors. In the divergence situation, beam width broadened, while it narrowed when in convergence. In the collimation situation (Figure 4b,c,f), beam width remained unchanged, showing a property of transverse localization. The beam-behavior-related property of the 2D PHC allows promising application in optical switch and optical lens when considering nonlinearity. In Figure 4b, the propagation of the SC wave showed that the wave vector was much larger than the reciprocal lattice vector inside the PHC. The subwavelength propagation of waves indicated that the proposed structure can overcome the diffraction limit.

**Figure 5:**
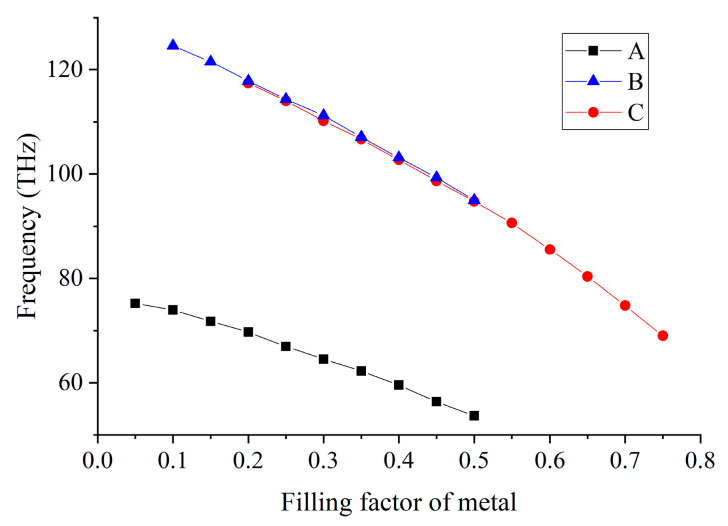
The frequencies of the SC points A (black), B (blue) and C (red) as functions of the filling factor (of metal) of the HMM.

The generation of the unique modes and twisted bands of the 2D PHC required the HMM to perform as a Type-I HMM. The positions (frequencies) of those SC points depended on the filling factor of HMM, as shown in Figure 5. The change of the self-collimation frequency of the twisted band was obtained by using the same method used in the calculation of the band structure. We applied different filling factors for the hyperbolic metamaterial, and calculated the model with different parameters multiple times, to obtain different band structures. Finally, we examined the frequencies of the degenerate points (SC points) of those bands. In such a way, we obtained the relation between the frequencies of SC points and the filling factor of the hyperbolic metamaterial. When the filing factor of HMM increased, the frequencies of SC modes decreased. This could be explained by the fact that, inside the HMM rods, the *x* components of the electric fields of the SC modes were much larger than the *y* components. Thus, for the effective dielectric tensors, only their *x* components were notable, and the increasing of the filling factor would increase $\varepsilon_{xx}(\varepsilon_{⊥})$, following the decreasing of frequencies of the SC modes. From Figure 5, it is also obvious that when the HMM no longer performed as a Type-I HMM, e.g., the filling factor of HMM was either too high or too low, the twisted bands would be covered by flat bands or no longer exist. The longitudinal and transverse components of permittivity of the HMM are two factors affecting the formation of the twisted bands. In various application situations, HMMs can be adjustable with proper filling factor to meet needs. Besides this, parameters, such as the lattice constants of the PC, also affect the Bloch modes of the PHC.

### 3.2. Switching Based on SC Effect

**Figure 6:**
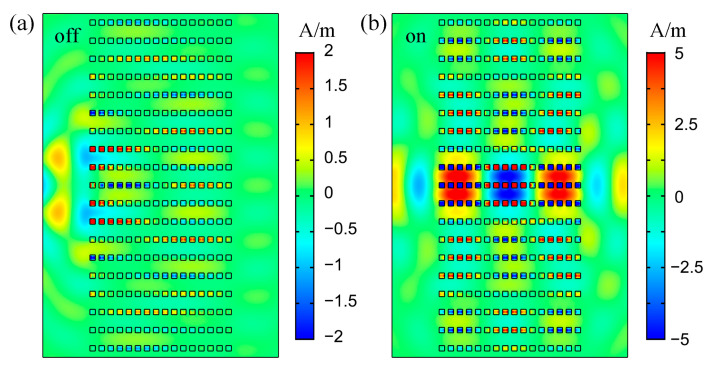
Magnetic field distributions of wave propagating in PHCs at 58.00 THz with pump powers of (**a**) 10^6^ V/m (approach linear regime) and (**b**) 3.4 × 10^6^ V/m (nonlinear regime).

HMMs have the advantage of providing large tunability for all-optical applications. When introducing nonlinearity for the dielectric layers of HMM, the effective permittivity of the HMM rods in the PHC can be changed by nonlinear effect, and further modify the property of the PHC. The Kerr effect changes the permittivity of dielectric layers as [31]

(4) $$\varepsilon_{\mathrm{NL}}=\varepsilon_{d}+\chi^{\left(3\right)}{\left|E\right|}^{2},$$

where *χ*^(3)^ is the third-order nonlinear optical susceptibility and *E* is the field intensity. In simulations, we set *χ*^(3)^ as 0.07 μm^2^/V^2^, which is achievable with semiconductor materials. We used the frequency domain study of COMSOL for the simulation of wave propagation. The effective permittivity of HMM rods was calculated by applying the nonlinear permittivity $\varepsilon_{\mathrm{NL}}$ for the dielectric layers, instead of the linear permittivity $\varepsilon_{d}$. In the PHC system, the permittivity change of dielectric layers would lead to huge change in the dispersion properties of Bloch modes, as well as in the twisted bands. As shown in Figure 6, considering an incident light with the frequency of 58.00 THz propagating in the nonlinear PHC, the beam behavior changed from divergence (“off”) to collimation (“on”) by increasing the pump power from 10^6^ V/m (approach linear regime) to 3.4 × 10^6^ V/m (nonlinear regime). The extinction ratio of such a switch was 10l g (*P*_on_/*P*_off_) = 19.75 dB. Note that the material loss of the Ag layers affected the transmittance of the system. However, with the small size model, the transmittance was not very poor. Transmittances of the PHC working on 59.52 THz, 102.6 THz and 102.9 THz were 31.9%, 9.6% and 23.7%, respectively. We applied 19 rows of HMM rods for the PHC, which maybe was not the most optimized structure for transmission of waves (because the self-collimation effect is affected by structure parameters). Only with a large scale of PHCs, would the loss be significant and unacceptable. In a bulk HMM, the loss of Ag would affect the performance of switching a lot. Our proposed PHCs can overcome the problem of high loss with Bloch modes, and thus can be superior than bulk HMMs. Moreover, in the “off” state, since the operating frequency is very near to the flat bands of the PHC, a large amount of light was reflected on the boundary, which further decreased the transmitted power and increased the extinction ratio. Changing the beam behavior did not require the transformation of HMM types. Thus, the efficiency and tunability of the PHC is higher than that of conventional infinite 1D HMM components. Moreover, in the convergence situation, the PHC may be utilized as an effective tunable lens, with focal length changing according to the incident power.

**Figure 7:**
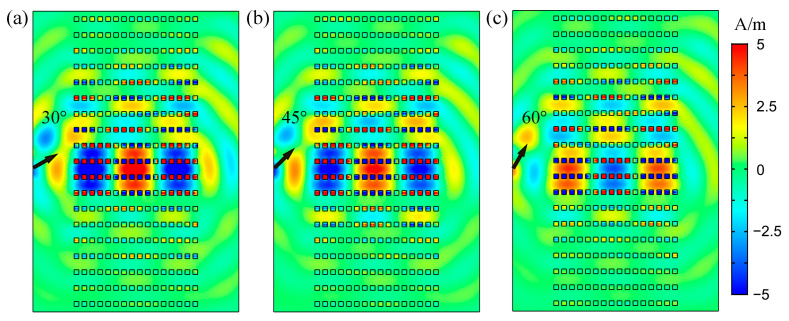
Magnetic field distributions of wave propagating in the PHC at 58.00 THz with pump power of 3.4 × 10^6^ V/m (nonlinear regime), the incident angles are (**a**) 30°, (**b**) 45° and (**c**) 60°.

As mentioned above, the SC modes in the twisted bands of PHC support incident light of different directions. Here, we demonstrated the “on” states of the switch when the angles between the incident light and PHC boundary were 30°, 45° and 60°, respectively, as shown in Figure 7. With various incident angles, the wave propagating still indicated strong field confinement, maintaining the SC effect.

The PHC switch can support a large number of ports, indicating huge potential in scalability. The PHC switching also has an advantage on switching time. Existing technologies of switches, based on MEMS or liquid crystals, require milliseconds of switching time. Conventional switches, based on resonant cavities, such as resonant rings or PC cavities, also require a longer time. The PHC switches, on the other hand, are based on the behavior of propagating waves. So, they can achieve a very short switching time. When the PHC switch is working on the “on” state, the SC effect can prevent energy loss by scattering, reducing its insertion loss and crosstalk. In summary, the comparison of the performance of different optical switching technologies is shown in Table 1, and the PHC switch indicates promising performance

### 3.3. PHCs with CaF_2_ and Si Matrices

**Figure 8:**
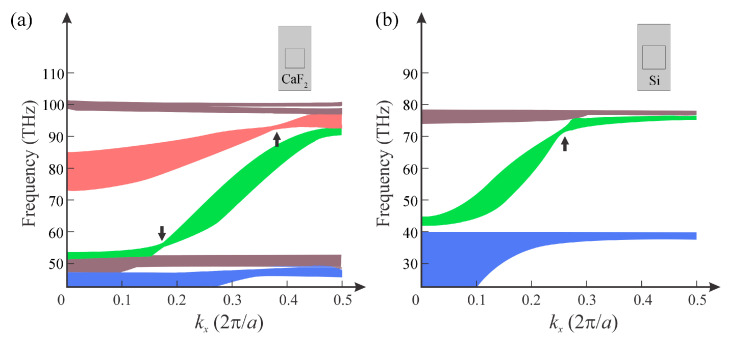
Band structures of the PHC with (**a**) CaF_2_ matrix and (**b**) Si matrix.

In reality, the manufacturing of the proposed systems is not feasible, as we have imagined a periodic array of parallelepipeds of thin layers of Si and Ag in an air matrix. This kind of system, although it presents large values of permittivity contrasted with an absence of absorption, is impossible to fabricate. In fact, by means of several real epitaxial and photolithographic methods it would be possible to implement such a kind of structure, but all would require the use of some matrix with permittivity larger than 1, and possibly, some degree of absorption. Here, we performed similar calculations by using CaF_2_ and Si as the matrix material. We performed calculations of the band structures by using matrices of CaF_2_ and Si, as shown in Figure 8a,b, respectively. We found the twisted-band property of the PHC still existed. In Figure 8a, we used experimental data of the refractive index of CaF_2_ [32]. The lattice constants of the PHC in *x* and *y* directions were *a* = 1 μm and *b* = 2*a* = 2 μm, respectively, and the side length of the HMM rods was *r* = 0.6*a*. The filling factor of the HMM rods changed to 0.5, with the thicknesses of the Ag and Si layers being *d*_m_ = *d*_d_ = 100 nm. In Figure 8b, we used experimental data of the refractive index of Si. The lattice constants of the PHC in *x* and *y* directions were *a* = 1 μm and *b* = 2*a* = 2 μm, respectively, and the side length of the HMM rods was *r* = 0.7*a*. The filling factor of the HMM rods changed to 0.7, with the thicknesses of the Ag and Si layers being *d*_m_ = 140 nm and *d*_d_ = 60 nm, respectively. We found that the PHC structure did not require the matrix to have low permittivity. Since the absolute value of the effective permittivity of HMM is very large, various materials, even those having a large permittivity, can be used as a matrix in the system, and the unique twisted bands could still be obtained.

### 3.4. Full Electromagnetic Calculations of PHCs

**Figure 9:**
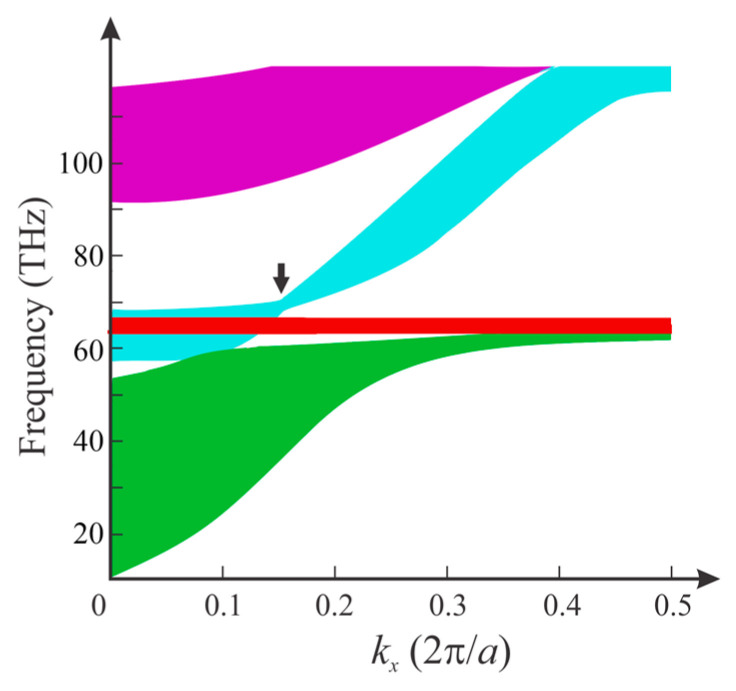
Band structure of the PHC by using full electromagnetic calculation.

Regardless of the need for a large amount of computing resources and time, we calculated the band structure of PHC by using the full electromagnetic calculation, and the result is shown in Figure 9. We used 20 layers of Ag and Si for the HMM rods, and other parameters remained the same as those in Section 3.1. In the figure, we can find the twisted band with a degenerate point, although the frequency of the degenerate point was different from the one obtained by using the effective permittivity of the HMM rods. Despite the accuracy, the result indicated that the phenomenon could be well predicted by the effective medium approximation [28,33], showing the validity of the approach.

## 4. Conclusions

In conclusion, we have proposed 2D PHCs containing finite-size Type-I HMM square rods, where the HMM rods consist of periodic layers of Ag layers and Si. We have numerically studied the dispersion property and band structure of the 2D PHC in the infrared region, and found the formation of twisted bands with SC points. Such twisted bands originate from the unique Bloch mode of the finite-size Type-I HMM rods. Dispersion properties of the PC change dramatically around the SC points. The SC frequencies changing in accordance with the filling factor of the HMMs was also investigated. Finally, by introducing nonlinearity to the system, switches based on the modification of beam behaviors by the 2D PHCs were investigated. Since the tunability of the system does not require the transformation of HMM type, it can provide large tunability. The 2D PHCs in the infrared region can be applied as all-optical high-performance switches or tunable lens.

## Figures and Tables

**Table 1:** Comparison of the performance of different optical switches.

	Switching Time	Insertion Loss	Crosstalk	Power Consumption
MEMS	10–20 ms	Low	Low	Medium
liquid crystal	100 ms	Medium	Medium	Low
SOA	~ns	Very low	Low	High
MZI	~ns	High	High	Medium
PHC *	~ns	Medium	Medium	Medium

* Based on theoretical analysis and simulation results.

## Data Availability

The data presented in this study are available on request from the corresponding author.

## Supplementary Materials

The following supporting information can be downloaded at: https://www.mdpi.com/article/10.3390/nano12121985/s1 [28,33] Figure S1: Eigenmodes solved by the full electromagnetic calculation and the approximated approach.

## References

[1] Poddubny A., Iorsh I., Belov P., Kivshar Y. (2013). Hyperbolic metamaterials. Nat. Photonics.

[2] Gric T., Hess O. (2018). Investigation of hyperbolic metamaterials. Appl. Sci..

[3] Davidovich M.V. (2019). Hyperbolic metamaterials: Production, properties, applications, and prospects. Physics-Uspekhi.

[4] Huo P.C., Zhang S., Liang Y.Z., Lu Y.Q., Xu T. (2019). Hyperbolic metamaterials and metasurfaces: Fundamentals and applications. Adv. Opt. Mater..

[5] Bhardwaj A., Srivastava K.V., Ramakrishna S.A. (2019). Enhanced coupling of light from subwavelength sources into a hyperbolic metamaterial fiber. J. Lightwave Technol..

[6] Cortes C.L., Newman W., Molesky S., Jacob Z. (2012). Quantum nanophotonics using hyperbolic metamaterials. J. Opt..

[7] Wang Z., Huo Y., Ning T., Liu R., Zha Z., Shafi M., Li C., Li S., Xing K., Zhang R. (2021). Composite structure based on gold-nanoparticle layer and HMM for surface-enhanced Raman spectroscopy analysis. Nanomaterials.

[8] Popov A.K., Myslivets S.A., Slabko V.V., Tkachenko V.A., George T.F. (2018). Shaping light in backward-wave nonlinear hyperbolic metamaterials. Photonics.

[9] Liu J., Chen W., Ma W., Chen Y., Deng X., Zhuang P., Ye Q. (2021). Biaxial hyperbolic metamaterial THz broadband absorber utilizing anisotropic two-dimensional materials. Results Phys..

[10] Feng K., Sivco D.L., Hoffman A.J. (2018). Engineering optical emission in sub-diffraction hyperbolic metamaterial resonators. Opt. Express.

[11] Azmoudeh E., Farazi S. (2021). Ultrafast and low power all-optical switching in the mid-infrared region based on nonlinear highly doped semiconductor hyperbolic metamaterials. Opt. Express.

[12] Lourtioz J.M., Benisty H., Berger V., Gerard J.M., Maystre D., Tchelnokov A. (2008). Photonic Crystals Towards Nanoscale Photonic Devices.

[13] Joannopoulos J.D., Johnson S.G., Winn J.N. (1995). Photonic Crystals: Molding the Flow of Light.

[14] Hou J., Zhou Y., Citrin D.S., Qiu X., Yang C., Chen S. (2021). Complete two-dimensional photonic bandgap in refractive-index ratio 2.1 photonic crystals due to high-order bands. Opt. Lett..

[15] He S., He Q., Wei L.F. (2021). Atomic-type photonic crystals with adjustable band gaps. Opt. Express.

[16] Narimanov E.E. (2014). Photonic hypercrystals. Phys. Rev. X.

[17] Smolyaninova V.N., Yost B., Lahneman D., Narimanov E.E., Smolyaninov I.I. (2014). Self-assembled tunable photonic hyper-crystals. Sci. Rep..

[18] Smolyaninov I.I. (2019). Nonlinear optics of photonic hyper-crystals: Optical limiting and hyper-computing. J. Opt. Soc. Am. B.

[19] Lu G., Zhou X.C., Zhao Y.P., Zhang K.Y., Zhou H.Y., Li J.Y., Diao C., Liu F., Wu A.L., Du G.Q. (2021). Omnidirectional photonic bandgap in one-dimensional photonic crystals containing hyperbolic metamaterials. Opt. Express.

[20] Pan T., Xu G.D., Zang T.C., Gao L. (2009). Goos-hanchen shift in one-dimensional photonic crystals containing uniaxial indefinite medium. Phys. Status Solidi B.

[21] Zheng Y., Wang Q., Lin M., Ouyang Z. (2022). Enhancement of self-collimation effect in photonic crystal membranes using hyperbolic metamaterials. Nanomaterials.

[22] Tsakmakidis K.L., Pickering T.W., Hamm J.M., Page A.F., Hess O. (2014). Completely stopped and dispersionless light in plasmonic waveguides. Phys. Rev. Lett..

[23] Guo Z., Jiang H., Chen H. (2020). Hyperbolic metamaterials: From dispersion manipulation to applications. J. Appl. Phys..

[24] Galfsky T., Gu J., Narimanov E.E., Menon V.M. (2017). Photonic hypercrystals for control of light-matter interactions. Proc. Natl. Acad. Sci. USA.

[25] Aspnes D.E., Studna A.A. (1983). Dielectric functions and optical parameters of Si, Ge, GaP, GaAs, GaSb, InP, InAs, and InSb from 1.5 to 6.0 eV. Phys. Rev. B..

[26] Shkondin E., Takayama O., Aryaee Panah M.E., Liu P., Larsen P.V., Mar M.D., Jensen F., Lavrinenko A.V. (2017). Large-scale high aspect ratio Al-doped ZnO nanopillars arrays as anisotropic metamaterials. Opt. Mater. Express.

[27] Vial A., Laroche T., Dridi M., Cunff L.L. (2011). A new model of dispersion for metals leading to a more accurate modeling of plasmonic structures using the FDTD method. Appl. Phys. A Mater. Sci. Process..

[28] Popov V., Lavrinenko A.V., Novitsky A. (2018). Surface waves on multilayer hyperbolic metamaterials: Operator approach to effective medium approximation. Rhys. Rev. B.

[29] Johnson P.B., Christy R.W. (1972). Optical constants of the noble metals. Phys. Rev. B.

[30] Babar S., Weaver J.H. (2015). Optical constants of Cu, Ag, and Au revisited. Appl. Opt..

[31] Yang G., Guan D.Y., Wang W.T., Wu W.D., Chen Z.H. (2004). The inherent optical nonlinearities of thin silver films. Opt. Mater..

[32] Malitson I.M. (1963). A redetermination of some optical properties of calcium fluoride. Appl. Opt..

[33] Popov V., Lavrinenko A.V., Novitsky A. (2016). Operator approach to effective medium theory to overcome a breakdown of Maxwell Garnett approximation. Phys. Rev. B.

