# Supplementary material for: Twisted Bands with Degenerate Points of Photonic Hypercrystals in Infrared Region
Source: Nanomaterials (Basel). 2022 Jun 9;12(12):1985. doi: 10.3390/nano12121985 (PMC9228899; doi:10.3390/nano12121985)
Supplement: Supplementary file 1 [file nanomaterials-12-01985-s001.zip › nanomaterials-1721425-supplementary.pdf]

Supplementary: Effective Medium Approximation

Twisted Bands with Degenerate Points of Photonic Hypercrystals in Infrared Region

Yaoxian Zheng [1], Qiong Wang [1], Mi Lin [1], Luigi Bibbò [2] and Zhengbiao Ouyang [1,*]

THz Technical Research Center of Shenzhen University, Shenzhen Key Laboratory of Micro-Nano Photonic Information Technology, Key Laboratory of Optoelectronic Devices and Systems of Ministry of Education and Guangdong Province, College of Physics and Optoelectronic Engineering, Shenzhen University, Shenzhen 518060, China; jewel5282@163.com (Y.Z.); qwang@szu.edu.cn (Q.W.); linfengas111@szu.edu.cn (M.L.)

[2] Department of Information Engineering, Infrastructure, and Sustainable Energy (DIIES), Mediterranea University of Reggio Calabria, Reggio Calabria 89124, Italy; luigi.bibbo@unirc.it

* Correspondence: zbouyang@szu.edu.cn

The conventional quasistatic approach needs to consider retardation effects, and is insufficient for the correct description of wave propagation in a system. However, the method we applied is a general nonlocal homogenization approach [28,33], which was developed by the adaption of finding the effective permittivity tensor of a multilayered structure. The approach considered spatial dispersion, and could avoid the breakdown of the effective medium approximation, and, as such, was found to be valid and effective.

We could use full electromagnetic calculation to examine the approach. We applied two kinds of setups. One was HMM rods containing Ag and Si layers, and full electromagnetic calculation was used, by means of the eigenvalue solver of COMSOL. The other one was a bulk HMM rod with effective permittivity considered, which contained no layers. We found that the approximated approach reached a similar result as that of the full electromagnetic calculation. Figure S1 shows the same eigenmode solved by the two methods. We used the full electromagnetic calculation for 6, 20 and 40 layer structures. The results indicated that, for the eigenvalue solver of COMSOL, effective medium approximation provided a valid and effective method for calculating the eigenmodes of the PHC system.

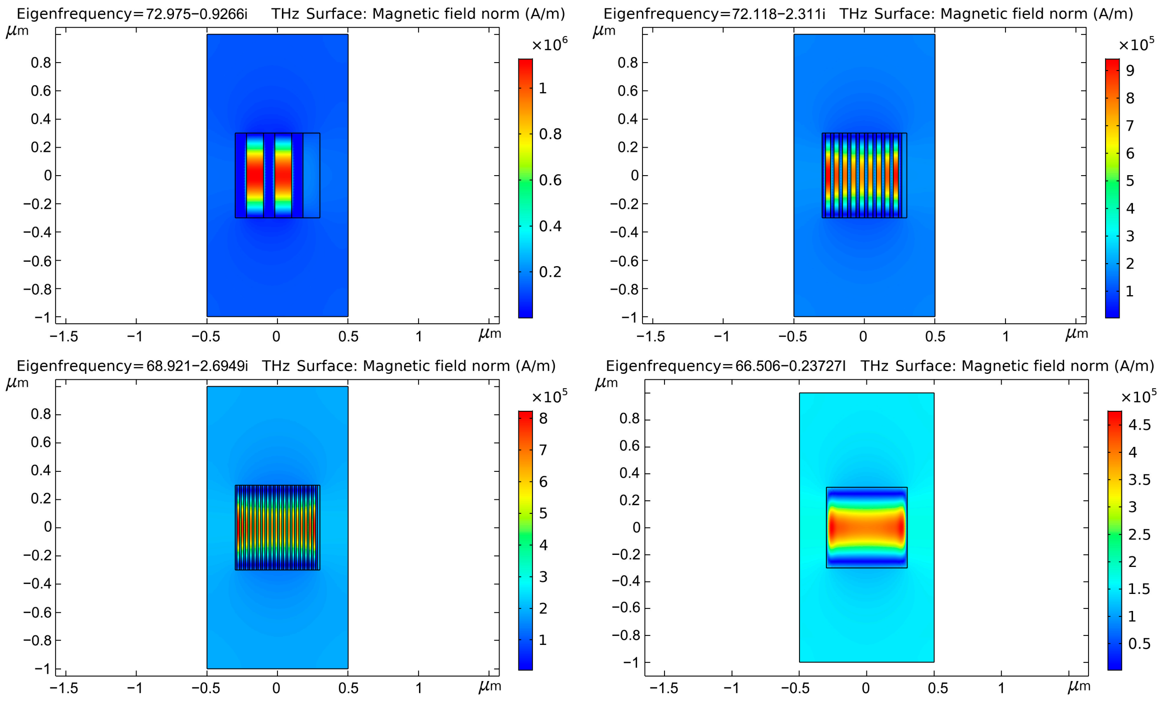

Figure S1. Eigenmodes solved by the full electromagnetic calculation and the approximated approach.

Figure S1 also indicates the lack of accuracy in calculations by the approximated approach, which can be improved by increasing the number of layers used.

References

[28] Popov, V.; Lavrinenko, A.V.; Novitsky, A. Surface waves on multilayer hyperbolic metamaterials: Operator approach to effective medium approximation. *Rhys. Rev. B* **2018**, *97*, 125428.

[33] Popov, V.; Lavrinenko, A.V.; Novitsky, A. Operator approach to effective medium theory to overcome a breakdown of Maxwell Garnett approximation. *Phys. Rev. B* **2016**, *94*, 085428.
